# Predictive value of nasopharyngeal microbiota for necrosis after re-irradiation in recurrent nasopharyngeal carcinoma

**DOI:** 10.1186/s12885-025-14842-1

**Published:** 2025-09-29

**Authors:** Kai Wen, Ze-Rong Huang, Yong-Long Liu, Wen-Bin Wu, Zi-Han Qin, Yan-Feng Ouyang, Xiong Zou, Rui You, You-Ping Liu, Ming-Yuan Chen, Yi-Jun Hua

**Affiliations:** 1 Department of Nasopharyngeal Carcinoma, Sun Yat-Sen University Cancer Center, 651 Dongfeng East Road, Guangzhou, 510060 People’s Republic of China; 2 State Key Laboratory of Oncology in South China, Guangdong Key Laboratory of Nasopharyngeal Carcinoma Diagnosis and Therapy, Sun Yat-Sen University Cancer Center, Guangzhou, 510060 People’s Republic of China; 3 Department of Ultrasound, State Key Laboratory of Oncology in South China, Collaborative Innovation Center of Cancer Medicine, Sun Yat-Sen University Cancer Center, 651 Dongfeng Road East, Guangzhou, 510060 People’s Republic of China; 4 Nasopharyngeal Cancer Center, Fifth Affiliated Hospital of Sun Yat-Sen University, 52 Meihua East Road, Zhuhai, Guangdong 519000 People’s Republic of China

**Keywords:** Nasopharyngeal Carcinoma, Recurrent, Re-irradiation, Nasopharyngeal Necrosis, Microbiota

## Abstract

**Background:**

Post-radiation nasopharyngeal necrosis (PRNN) is a severe complication following re-irradiation in patients with recurrent nasopharyngeal carcinoma (NPC). This study aimed to explore the association between nasopharyngeal microbiota and PRNN in patients with recurrent NPC undergoing re-irradiation and to evaluate the predictive value of the microbiota for PRNN.

**Methods:**

This retrospective study collected data from 113 patients with recurrent NPC who underwent re-irradiation at the Sun Yat-sen University Cancer Center (SYSUCC) between January 2020 and November 2022. Patients were divided into necrosis and non-necrosis groups based on the development of necrosis after re-irradiation. 5R 16S rRNA sequencing of nasopharyngeal biopsy tissues conducted before re-irradiation was used to assess microbiota composition, diversity, and functional predictions. Clinical features and selected microbial markers were used in a random forest model to predict the occurrence of PRNN.

**Results:**

Of the 113 patients with recurrent NPC who underwent re-irradiation, 60 developed PRNN, while 53 did not. Proteobacteria and Firmicutes were the dominant phyla in the nasopharyngeal microbiota of all the patients with recurrent NPC. The necrosis group exhibited significantly higher alpha diversity and distinct beta diversity than the non-necrosis group did. A predictive model that combined clinical features (gross tumor volume [GTV]) with microbiome characteristics achieved an AUC of 87.9% in the training set and 86.9% in the test set, demonstrating robust predictive performance.

**Conclusions:**

Nasopharyngeal microbial diversity prior to re-irradiation was significantly higher in the necrosis group. Our predictive model, integrating clinical and microbial features, demonstrated strong performance in predicting PRNN and offers a promising tool for early intervention and prevention strategies.

**Supplementary Information:**

The online version contains supplementary material available at 10.1186/s12885-025-14842-1.

## Background

In the southern regions of China, nasopharyngeal carcinoma (NPC) is a prevalent form of head and neck cancer [1]. Despite advancements in radiation dosimetry and increased dose intensity with the introduction of intensity-modulated radiotherapy (IMRT), local recurrence still affects 10–20% of patient [2–4]. For recurrent NPC, endoscopic surgery is an option for patients with resectable tumors; however, its feasibility is often compromised by the involvement of critical anatomical structures surrounding the nasopharynx [5, 6]. High-dose re-irradiation remains the only curative treatment option for patients with unresectable tumors [7].

For patients with recurrent NPC undergoing re-irradiation, post-radiation nasopharyngeal necrosis (PRNN) is one of the most severe late adverse events [8]. It involves necrosis of tissues surrounding the nasopharynx, including the mucosa, longus capitis muscle, parapharyngeal tissues, and skull base [9], and is often accompanied by symptoms such as headache, foul odor, purulent debris, and bacterial infections, which significantly impact patients'quality of life [10]. PRNN can lead to life-threatening bleeding if inadequately managed [11], with mortality rates as high as 72.7% when the internal carotid artery (ICA) is exposed to necrotic lesions [12].

Previous rigorous studies have identified the accumulated total prescription dose to the gross tumor volume (GTV) and volume of recurrent tumors as significant predictors of PRNN development [13]. A recent randomized phase 3 clinical trial demonstrated that hyperfractionated IMRT could significantly decrease the rate of PRNN [7]. However, the exact biological and molecular mechanisms underlying PRNN remain unclear.

Nasopharyngeal infections play a critical role in PRNN development [14–16]. Recent studies have shown that microbiota are present within NPC tissues, with the nasopharynx being the primary origin of NPC intratumoral bacteria [17]. The microbiota is regarded as an invisible organ that modulates numerous physiological functions, and dysbiosis has been implicated in various diseases [18]. In radiation-induced injury, dysbiosis may enhance cytokine expression and contribute to disease progression [19]. Furthermore, studies have demonstrated that the intratumoral bacterial load in newly diagnosed NPC serves as a reliable prognostic biomarker [20].

However, little is known about the association between the intratumoral microbiota and recurrent NPC. To better understand the role of intratumoral microbiomes and their interaction with PRNN, this retrospective study aimed to develop and validate a predictive model based on clinical and microbial characteristics to assess PRNN risk following curative re-irradiation for recurrent NPC.

## Methods

### Study population

We retrospectively reviewed the data of 146 patients diagnosed with recurrent NPC who underwent re-irradiation between January 2020 and November 2022 at the Sun Yat-sen University Cancer Center (SYSUCC). The inclusion criteria were as follows: (1) age between 18 and 70 years; (2) histopathologically confirmed locally recurrent NPC; (3) received re-irradiation at Sun Yat-sen University Cancer Center; and (4) complete medical history records, imaging, and laboratory examinations available at the time of recurrent diagnosis and during treatment, with regular follow-up at Sun Yat-sen University Cancer Center. Exclusion criteria were: (1) presence of distant metastasis and (2) concurrent nasopharyngeal necrosis at the time of recurrence diagnosis.

After the review, 113 patients with NPC met the inclusion and exclusion criteria. Data on survival, recurrence, distant metastasis, and mortality were collected from medical records and follow-up assessments after re-irradiation.

This study was approved by the Ethics Committee of the Sun Yat-sen University Cancer Center. As a retrospective analysis, the need for informed consent for the use of archived tissue samples was waived. Patient confidentiality was maintained, and all data were anonymized before analysis.

### Clinical data

Based on previous studies, we collected potential clinical characteristics related to PRNN from hospitalization records, including demographic factors (sex and age), medical history (allergy history, smoking history, alcohol consumption, diabetes mellitus history), physical metrics (Body Mass Index [BMI], Karnofsky Performance Status [KPS] score), tumor staging information (initial T-stage, initial N-stage, clinical stage, recurrent T-stage, recurrent N-stage, recurrent clinical stage), interval of recurrence, treatment details (concurrent chemotherapy or immunotherapy during re-irradiation, initial radiation dose and fractionation, recurrent GTV re-irradiation prescription dose and fractionation, duration of re-irradiation, accumulated total prescription dose [initial and recurrent]), and hematological and biochemical results, including white blood cell count (WBC#), neutrophil percentage (NEUT%), neutrophil count (NEUT#), red blood cell (RBC) count, hemoglobin (HGB), and C-reactive protein (CRP) levels.

### Second-course radiotherapy

All the patients were treated with IMRT. The GTV, clinical target volume (CTV), and organs at risk (OARs) were contoured slice-by-slice on CT images. The GTV was delineated based on fiberoptic nasopharyngoscopy, planning CT, MRI, and PET-CT findings. The CTV was the GTV with a 5 mm margin (2 mm margin posteriorly), and the planning target volume was formed from the gross tumor volume and the clinical target volume with a 3 mm margin (1 mm margin posteriorly). For patients who were node-positive, the gross nodal tumor volume of the cervical lymph nodes (GTVnd) was outlined, and a 3 mm margin was added to form the planning target volume.

Radiotherapy dose data were retrieved from the treatment planning system for further dose feature extraction. The prescribed doses were 60 Gy for the PTV derived from the GTV, 60 Gy for the PTV of the GTVnd (if node-positive), and 54 Gy for the PTV of the CTV, delivered in 27 fractions, once daily. These dose specifications were based on findings from a prospective clinical trial conducted at the SYSUCC [21]. All patients received full-course IMRT with 6-MV x-rays generated by a Trilogy linear accelerator (Varian Medical Systems, Palo Alto, CA, USA). Dose verification was performed before re-irradiation. The dose error between the measurement and the plan was less than 2%. For patients who received concurrent chemotherapy, cisplatin was administered at 100 mg/m^2^ every 3 weeks for two cycles during the course of re-irradiation.

### Follow-up duration

Follow-up duration was defined as the time from completion of re-irradiation to the date of the last clinical follow-up (including symptom evaluation, nasopharyngoscopy, or MRI), measured in months. The distribution of follow-up durations in the necrosis and non-necrosis groups was compared using the Mann–Whitney U test.

### Diagnosis of nasopharyngeal necrosis

The diagnosis of PRNN was primarily confirmed through endoscopic evaluation, followed by biopsy, which is considered the gold standard. For patients in whom biopsy was not feasible, diagnosis relied on specific MRI features, including discontinuous nasopharyngeal mucosa lines and soft tissue defects without contrast enhancement, combined with clinical symptoms such as foul nasal odor, refractory headache, necrotic tissue, and skull base osteoradionecrosis observed in the nasopharyngeal cavity during endoscopy [22]. All patients underwent standardized follow-up visits every three months during the first two years after re-irradiation and every six months thereafter. Each follow-up included clinical assessment, nasopharyngoscopy, and MRI to monitor for PRNN. The radiological assessments were performed and interpreted by professional radiation oncologists.

### Tissue collection

Nasopharyngeal biopsy tissue was collected from 113 patients with recurrent NPC. Each biopsy tissue sample was paraffin-embedded and placed in a sterile, nucleus-free cryopreservation tube. To serve as a negative control for 16S rRNA gene sequencing, a corresponding blank collection tube was prepared, left open in the collection area for 30 s, and immediately stored alongside the sample tubes.

### DNA/RNA extraction, amplification, sequencing

DNA and RNA were extracted from nasopharyngeal carcinoma biopsy tissues using the AllPrep DNA/RNA Micro Kit, QIAamp DNA FFPE Tissue Kit, or QIAGEN DNeasy PowerSoil Kit (QIAGEN GmbH). Sterile pipettes, pipette tips, and nonenzymatic kit components were UV-irradiated for at least 1 h prior to use.

Genomic DNA were extracted with the EZNA® Soil DNA kit in accordance with the manufacturer’s guidelines (D4015, Omega, Inc., USA) and stored immediately at − 80 °C for amplification.The 16S rRNA gene V3-V4 region was amplified with slightly modified microbiome primers (515F-805 R) in a two-step PCR protocol [23]. To analyze the intratumor microbiota, a modified 5 region amplification of the16S rRNA gene amplification was performed [24]. The amplification products were purified using AMPure XT beads (Beckman Coulter, USA) and quantified using Qubit (Invitrogen, USA). Subsequently, the amplicon pools were sequenced and the quantity and size of the amplified libraries were evaluated using an Agilent 2100 Bioanalyzer (Agilent, USA). Samples were sequenced on the Illumina NovaSeq platform according to the manufacturer’s recommendations, which were supported by Lc-Bio Technologies Co., Ltd. (Hangzhou, China). Subsequently, raw reads were analyzed using QIIME2 software, and quality filtration was performed using fqtrim software (V.0.9.4) to acquire high-quality clean tags. DABA2 software was used to construct the sequence and feature tables containing amplicon sequence variants (ASVs). Taxonomic analysis for species annotation was conducted using BLAST with the SILVA and NT-16S databases. MAFFT software was used to identify the predominant species in different groups and diverse sequence alignments.

Alpha diversity was assessed using the Chao1 and Shannon indices in QIIME2, while beta diversity was characterized by principal component analysis (PCA) and principal coordinate analysis (PCoA) with R packages ade4 and vegan.

### Statistical analyses of microbial data

Continuous variables were expressed as mean ± standard error of the mean (SEM) or median with interquartile range (IQR), while categorical data were described as frequencies. Differences in alpha diversity were verified using Wilcoxon signed-rank test. Alpha diversity differences were assessed using the Wilcoxon test, and beta diversity, reflecting differences in sample community composition, was analyzed via PCA and PCoA using the weighted UniFrac index. The relationship between community composition and microbiome extrinsic elements was analyzed using permutational multivariate analysis of variance (PERMANOVA) and the VEGAN R packages.

Differences in microbial abundance were analyzed using the Wilcoxon rank-sum test in the STAMP software. Statistically enriched microorganisms were identified using the linear discriminant analysis effect size (LEfSe) algorithm, with an LDA score > 3 and Bonferroni-adjusted *p* < 0.05.

### Features selection and model construction

Univariate and multivariate logistic regression analyses were used to identify and distinguish independent clinical risk factors. This was followed by an analysis of differentially abundant ASVs, in which key microbiota features showing significant differences (*p* < 0.05) between the two groups were identified. Microbiota features with a linear discriminant analysis (LDA) value greater than three were further classified as microbiota biomarkers.

For predictive modeling, the dataset was randomly divided into training and testing cohorts at a 6:4 ratio. Random forest models with stratified fivefold cross-validation were employed to predict PRNN. Two models were established based on different feature sets: (1) the Microbiota Model, which included only microbiological features, and (2) the GTV-Microbiota Model, which combined clinical variables with microbiological features. The performance of each model was assessed using Receiver Operating Characteristic (ROC) curves and the Area Under the Curve (AUC) metric. Model comparisons were performed using the DeLong test to evaluate statistical differences in predictive performance.

### Prediction of pathway composition by PICRUSt2

Metagenomic functional composition from the taxa abundance was inferred using Phylogenetic Investigation of Communities by Reconstruction of Unobserved States (PICRUSt2) [25]. An amplicon sequence variant (ASV) table was generated, where the taxonomy of each ASV was assigned using the Kraken 2 classification against the Silva database version 138. The abundance of MetaCyc pathways was estimated using PICRUSt2 with default options. Pathways with a relative abundance of < 0.01% were converted to 0 to reduce noise. Python version 3.6.2 and R version 4.0.4. were used for all statistical analyses.

## Results

### Patient characteristics

This study included 113 patients with recurrent NPC, comprising 60 patients in the necrosis group and 53 in the non-necrosis group, categorized based on the occurrence of PRNN following re-irradiation. The baseline demographics and clinical characteristics of the patients in the two groups are summarized in Table 1. The median follow-up duration was 21.7 months (IQR, 12.5–30.8) in the non-necrosis group and 17.6 months (IQR, 12.9–27.9) in the necrosis group, with no significant difference between groups (p = 0.595) (Figure S1). The median age was 49 years (interquartile range [IQR]: 39–57), and 74 patients (65.5%) were male. All participants underwent IMRT and received a total dose of 60 Gy delivered in 27 fractions. Among the 113 patients, 3 (2.7%) had a history of diabetes mellitus. The GTV was the only parameter that showed a statistically significant difference between the two groups, whereas no significant differences were observed for other parameters.

**Table 1:** Distribution of demographic and clinical characteristics of patients in the non-necrosis and necrosis groups

Characteristic	Non-necrosis (*N* = 53)	Necrosis (*N* = 60)	*P* value
**Gender**			
Male	35 (66.0)	39 (65.0)	1
Female	18 (34.0)	21 (35.0)	
Age^*^	48.00 [39.00, 56.00]	49.00 [38.00, 58.25]	0.721
**Pathology**			0.82
I	4 (7.5)	3 (5.0)	
II	2 (3.8)	3 (5.0)	
III	47 (88.7)	54 (90.0)	
**Allergy history**	3 (5.7)	2 (3.3)	0.664
**Smoking history**	15 (28.3)	13 (21.7)	0.551
**Diabetes history**	2 (3.8)	1 (1.7)	0.599
**Alcohol history**	5 (9.4)	8 (13.3)	0.724
BMI^*^	22.00 [20.00, 24.00]	21.50 [19.00, 24.00]	0.238
KPS score^*^	90.00 [90.00, 90.00]	90.00 [90.00, 90.00]	0.812
GTV^*^	28.80 [22.30, 44.40]	41.10 [30.03, 62.20]	0.001
**Initial T stage**^**a**^			0.919
TI	1 (2.5)	2 (4.1)	
TII	5 (12.5)	7 (14.3)	
TIII	15 (37.5)	20 (40.8)	
TIV	19 (47.5)	20 (40.8)	
**Initial N stage**^**a**^			0.146
N0	2 (5.0)	7 (14.3)	
NI	15 (37.5)	19 (38.8)	
NII	20 (50.0)	15 (30.6)	
NIII	3 (7.5)	8 (16.3)	
**Initial clinical stage**^**a**^			0.677
I	0 (0.0)	1 (2.0)	
II	2 (5.0)	4 (8.2)	
III	17 (42.5)	17 (34.7)	
IV	21 (52.5)	27 (55.1)	
**Recurrent T stage**			0.731
TI	1 (1.9)	1 (1.7)	
TII	2 (3.8)	2 (3.3)	
TIII	30 (56.6)	28 (46.7)	
TIV	20 (37.7)	29 (48.3)	
**Recurrent N stage**			0.386
N0	35 (66.0)	37 (61.7)	
NI	14 (26.4)	21 (35.0)	
NII	2 (3.8)	2 (3.3)	
NIII	2 (3.8)	0 (0.0)	
**Recurrent stage**			0.912
I	1 (1.9)	1 (1.7)	
II	2 (3.8)	2 (3.3)	
III	28 (52.8)	28 (46.7)	
IV	22 (41.5)	29 (48.3)	
Radiotherapy interval (year) *	2.64 [1.95, 3.67]	2.80 [1.89, 5.13]	0.309
Follow-up duration (months)*	21.7 [12.5, 30.8]	17.6 [12.9, 27.9]	0.595
**Chemotherapy during re-irradiation**	40 (75.5)	42 (70.0)	0.66
**Anti-PD1 during re-irradiation**	20 (37.7)	19 (31.7)	0.632
**Anti-PDL1 during re-irradiation**	2 (3.8)	7 (11.7)	0.231
WBC *	5.92 [5.30, 6.72]	5.82 [4.76, 6.81]	0.359
NEUT% *	69.50 [66.40, 74.40]	71.30 [64.02, 75.60]	0.908
NEUT# *	4.22 [3.68, 4.99]	4.13 [3.13, 4.72]	0.269
RBC *	4.68 [4.17, 5.12]	4.62 [4.24, 5.00]	0.782
HGB *	137.00 [123.00, 148.00]	134.00 [118.75, 144.00]	0.353
CRP *	2.59 [1.03, 6.26]	2.60 [1.47, 6.84]	0.474

Note. —Unless otherwise specified, data are numbers of participants, with percentages in parentheses. BMI = body mass index, KPS = Karnofsky Performance Status, GTV = Gross Tumor Volume, PD1 = programmed cell death protein 1, PDL1 = Programmed cell death 1 ligand 1, WBC = white blood cell, NEUT% = Neutrophil ratio, NEUT# = Absolute Neutrophil Count, RBC = red blood cell, HGB = hemoglobin, CRP = C-reactive protein

^*^ Data are medians, with IQRs in square brackets

^**a**^Some patients lacked information on the initial treatment stage

### Exploring the potential clinical features to predict necrosis

To identify independent clinical risk factors for PRNN, potential clinical features were first analyzed using univariate logistic regression. Variables with a univariate analysis P-value < 0.2 were subsequently included in the multivariate logistic regression analysis. In the multivariate analysis, the adjusted odds ratios (AOR) for sex and hemoglobin were 2.73 (95% CI: 0.74–10.11, P = 0.132) and 0.99 (95% CI: 0.96–1.04, P = 0.796), respectively. The adjusted odds ratio for GTV of the recurrent tumor was 1.03 (95% CI: 1.01–1.06, P = 0.018), indicating that GTV is an independent risk factor for PRNN after re-irradiation. Detailed results of the regression analysis for all potential clinical variables are presented in Table 2.

**Table 2:** Univariate and Multivariate Analysis of Clinical Variables for Predicting Post-Radiation Nasopharyngeal Necrosis

Characteristic	Univariate Analysis OR (95% CI)	P	Multivariate Analysis aOR (95% CI)	aP
Age	1.02 (0.98—1.06)	0.389		
Gender	2.15 (0.81—5.72)	0.126	2.73 (0.74—10.11)	0.132
BMI	0.94 (0.82—1.07)	0.34		
Allergies History	0.30 (0.03—3.01)	0.306		
Smoking History	0.61 (0.22—1.74)	0.36		
Alcohol History	0.94 (0.22—4.08)	0.939		
GTV Volume	1.04 (1.01—1.06)	0.003	1.03 (1.01—1.06)	0.018
WBC	0.95 (0.76—1.19)	0.664		
NEUT%	0.98 (0.92—1.04)	0.45		
NEUT#	0.95 (0.74—1.21)	0.658		
HGB	0.97 (0.95—1.00)	0.065	0.99 (0.96—1.04)	0.796
RBC	0.87 (0.46—1.66)	0.673		
C-Reactive Protein	1.02 (0.99—1.06)	0.227		
Immunotherapy during re-irradiation	2.00 (0.34—11.61)	0.44		
Radiotherapy Interval	1.05 (0.94—1.17)	0.391		
Recurrent T Stage	1.25 (0.30—1.82)	0.505		
Recurrent N Stage	2.45 (0.21—28.89)	0.475		

### Nasopharyngeal microbiota composition in recurrent NPC

**Fig. 1:**
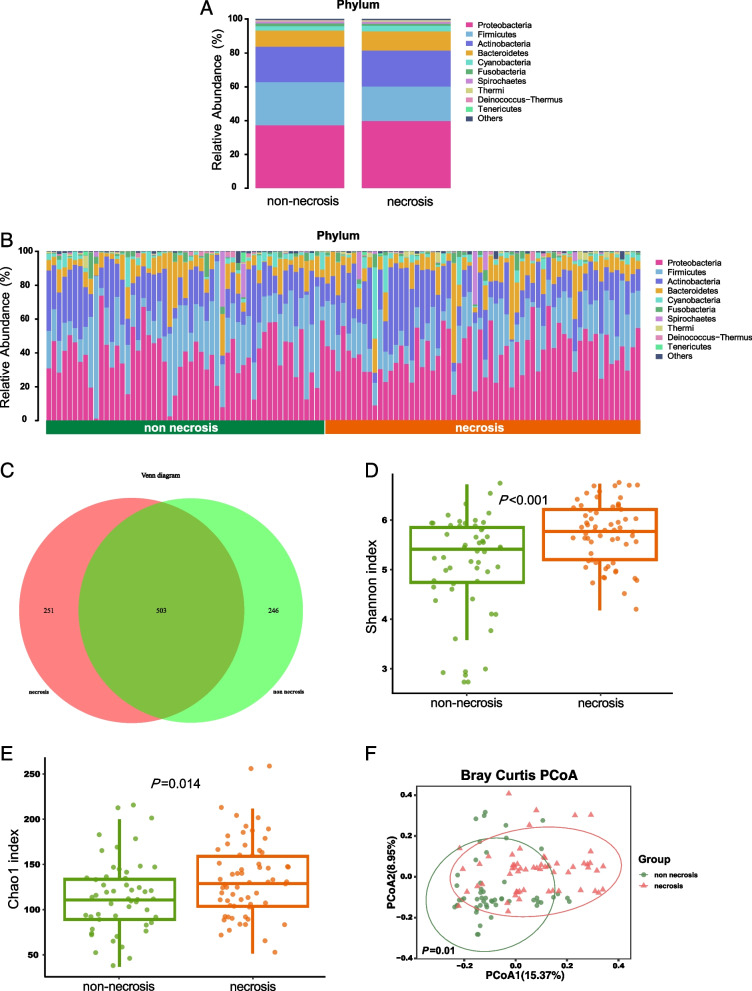
The nasopharyngeal microbiome in non-necrosis group significantly differed from that in necrosis group. **A** Stacked bar plots depicting the common taxa at the phylum level in two groups. **B** The relative abundances of nasopharyngeal microbes at the phylum level of non-necrosis group and necrosis group based on the species annotated by ASV. The 10 most abundant phyla of each group are shown. **C** The Venn diagram displayed the overlaps between two groups based on ASVs level. **D** and **E** Shannon (**D**) and Chao1 (**E**) indices obtained by ASV of 16S rRNA (*n* = 53 for the non-necrosis group and n = 60 samples for the necrosis group. **F** Principal coordinates analysis (PCoA) of all fecal samples from LARC patients using Bray–Curtis distance at the ASV level

First, we analyzed the main bacterial composition in the two groups across different taxonomic levels. At the phylum level, the nasopharyngeal microbiota was predominantly composed of Proteobacteria and Firmicutes, followed by Actinobacteria, Bacteroidetes, Cyanobacteria, Fusobacteria, and Spirochaetes (Fig. 1A). We compared the bacterial abundance between the two groups at the phylum level, which revealed considerable variability in the microbiota composition across samples in each group (Fig. 1B). A Venn diagram was used to illustrate the distribution of the bacterial genera between the two groups. Of the 1000 total genera identified, 503 genera were shared by both groups, while 251 genera were unique to the necrosis group (Fig. 1C).

### Bacterial diversity differences between necrosis and non-necrosis groups

To evaluate differences in bacterial diversity between groups, alpha diversity was assessed based on sequence alignment. The results showed that nasopharyngeal microbial alpha diversity indicated by Shannon and Chao1 indices was significantly higher in the necrosis group than in the non-necrosis group (Fig. 1D and 1E). Beta diversity was calculated using the Bray–Curtis method to illustrate the microbiome composition differences between the groups, and PCoA was conducted. PERMANOVA revealed a significantly distinct distribution of microbial communities between the two groups (Fig. 1F).

### Phylogenetic profiles of nasopharyngeal microbial communities

To identify differentially abundant taxa between the necrosis group and non-necrosis groups, LDA coupled with LEfSe was performed on the nasopharyngeal microbiota composition based on 16S rRNA gene sequencing.

**Fig. 2:**
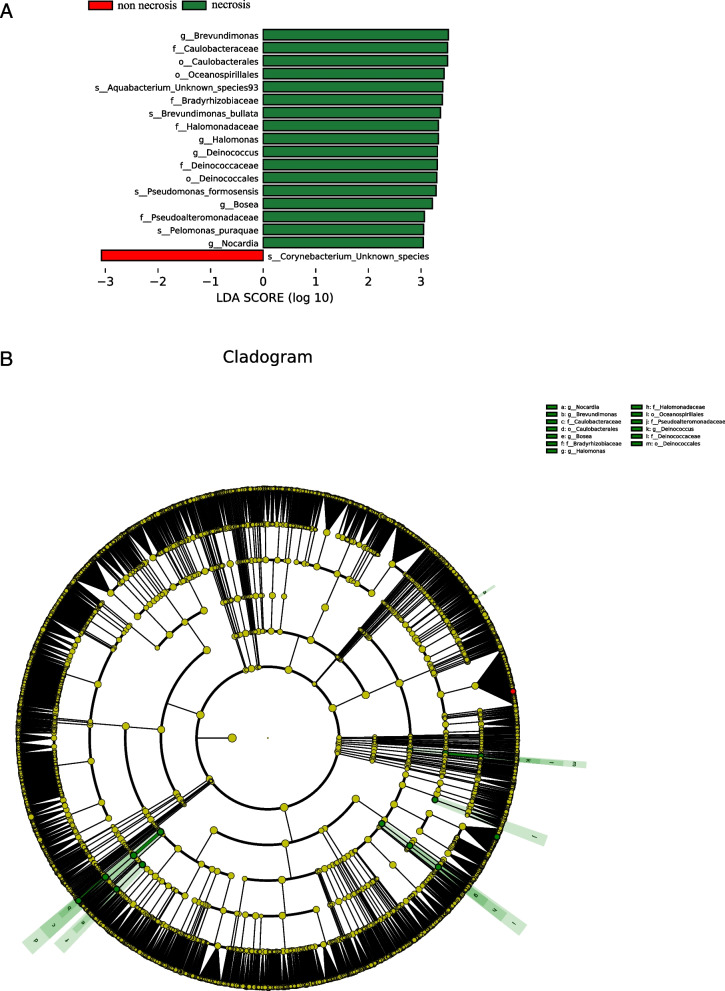
Differential abundance of nasopharyngeal microbial taxa between necrosis and non-necrosis groups (A and B) Differentially abundant taxa between non-necrosis and necrosis groups analyzed by LEfSe are projected as histogram (**A**) and cladogram (**B**). All listed taxa were significantly (Kruskal–Wallis test, *p* < 0.05; LDA score > 2) enriched for their respective groups (necrosis, green and non-necrosis, red)

A total of 73 bacterial taxa showed significantly different relative abundances between the two groups (LDA score > 2.0, *p* < 0.05). Among these, the taxa that were significantly enriched in the necrosis group included *g_Brevundimonas, f_Caulobacteraceae, o_Caulobacterales, o_Oceanospirillales, s_Aquabacterium_Unknown_species93, f_Bradyrhizobiaceae, s_Brevundimonas_bullata, f_Halomonadaceae, g_Halomonas, g_Deinococcus, f_Deinococcaceae, o_Deinococcales, s_Pseudomonas_formosensis, g_Bosea, f_Pseudoalteromonadaceae, s_Pelomonas_puraquae g_puraquae.* In contrast, *s_Corynebacterium_Unknown_species* was predominantly associated with the non-necrosis group (Fig. 2A and B).

### Prediction of gene function in the nasopharyngeal microbiota

In this study, we utilized the Phylogenetic Investigation of Communities by Reconstruction of Unobserved States (PICRUSt2) method to compare microbial gene functions between the necrosis and non-necrosis groups. Functional predictions were analyzed across multiple databases, including Clusters of Orthologous Genes (COGs), Kyoto Encyclopedia of Genes and Genomes (KEGG), KEGG orthology (KO), protein families (PFAM), and The Institute for Genomic Research Protein Families (TIGRFAM), which feature curated multiple sequence alignments.

**Fig. 3:**
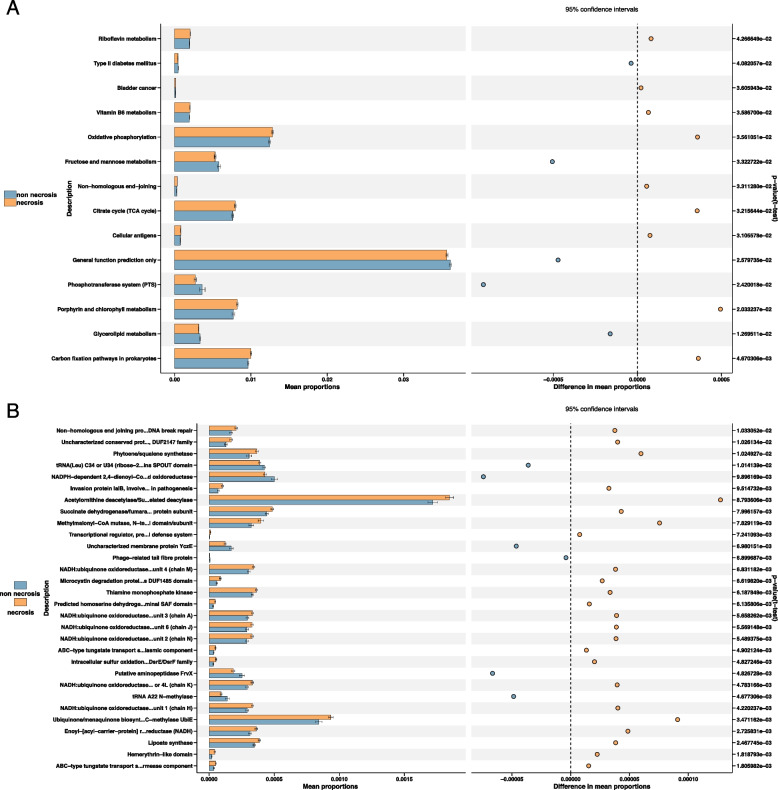
Prediction of gene function in the nasopharyngeal microbiota. **A** and **B** Predicted function of nasopharyngeal microbiota based on KEGG (**A**) and COGs (**B**) pathway analysis. The extended error bar plot showed the significantly different KEGG (**A**) and COGs (**B**)pathways between non-necrosis and necrosis group

Key functional differences were also identified. Within the KEGG pathways, notable functions included *Carbon fixation pathways in prokaryotes*, *Glycerolipid metabolism*, and *Porphyrin and chlorophyll metabolism* (Fig. 3A). In the COG database, important functions, such as *ABC-type tungstate transport system*, *Lipoate synthase*, and *Enoyl-[acyl-carrier-protein] reductase (NADH)* were significantly enriched (Fig. 3B).

### Clinical and microbiota models for predicting nasopharyngeal necrosis

A total of 73 ASVs identified through LEfSe analysis demonstrated the predictive potential of PRNN. To evaluate the predictability of the nasopharyngeal microbiome, these ASVs were used as input features to construct the PRNN prediction model. The 113 patients were randomly divided into two cohorts: 36 necrosis and 31 non-necrosis cases in the training cohort and 24 necrosis and 22 non-necrosis cases in the test cohort. After five repeats of fivefold cross-validation, 18 microbial variables were identified as the optimal feature set for model construction.

**Fig. 4:**
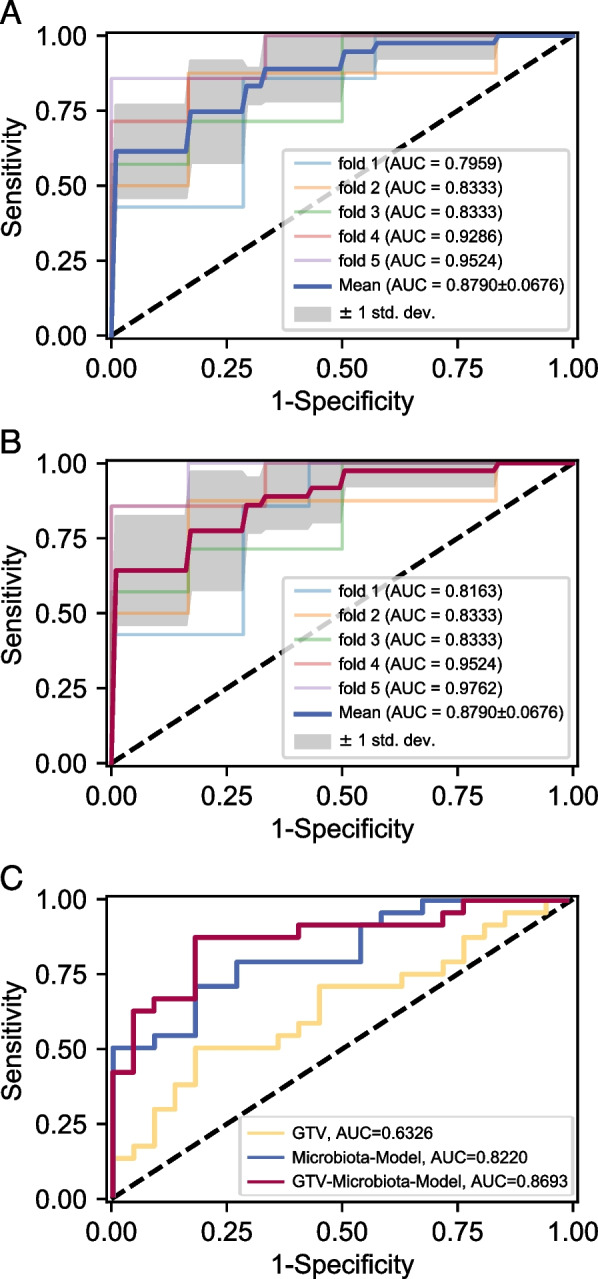
Predictive performance of different models using nasopharyngeal microbiome and clinical features. **A** Performance of the Microbiota model in the training cohort. The model, constructed using 18 microbial variables selected through 5 repeats of fivefold cross-validation, demonstrated an AUC of 87% (95% CI, 81–93%). **B** Performance of the GTV-Mcrobiota model in the training cohort. The model, which included 18 microbial variables and GTV, achieved an AUC of 88% (95% CI, 81–95%). **C** Performance of the models in the test cohort. The Microbiota model achieved an AUC of 82.2% (95% CI, 70.3–94.1%), while the combined GTV-Microbiota model achieved an AUC of 86.9% (95% CI, 76.3–97.5%). The GTV alone is also included for comparison

The Microbiota model showed a strong performance in the training cohort, achieving an AUC of 87% (95% CI, 81–93%) (Fig. 4A). Besides using differential ASVs as key metrics, the GTV-Microbiota model incorporated 18 ASVs and the clinical feature GTV as input features, achieving an AUC of 88% (95% CI, 81–95%) in the training cohort (Fig. 4B). In the test cohort, the Microbiota model achieved an AUC of 82.2% (95% CI, 70.3–94.1%), while the GTV-Microbiota model outperformed it with an AUC of 86.9% (95% CI, 76.3–97.5%) (Fig. 4C). Statistical comparisons using the DeLong test confirmed that the GTV-Microbiota model significantly outperformed the Microbiota model in predicting necrosis (Table 3).

**Table 3:** AUCs of GTV, Microbiota-Model and GTV-Microbiota Model in test set

	**AUC (95%CI)**	***P* value **^**†**^
GTV	0.6325 (0.4689, 0.7963)	–
Microbiota Model	0.8220 (0.7032, 0.9408)	0.0532
GTV-Microbiota Model^*^	0.8693 (0.7634, 0.9752)	0.0013

Abbreviations: AUC = area under the receiver operating characteristic curve, GTV = Gross Tumor Volume

^†^ *P* value was calculated with the DeLong test

^*^ The GTV-Microbiota-Model includes GTV and Microbiota variables

## Discussion

In this retrospective study, we demonstrated the clinical significance of intratumoral microbiota in predicting PRNN in patients with recurrent NPC undergoing re-irradiation. By comparing the necrosis group—patients who developed PRNN after re-irradiation—with the non-necrosis group, we identified significant differences in microbial composition between the two cohorts, with the necrosis group showing higher microbial diversity. These findings suggest a complex interaction between intratumoral microbiota, including genera such as *Brevundimonas*, *Caulobacteraceae*, *Deinococcus*, and *Halomonas*, and the tumor microenvironment, potentially contributing to PRNN development. Utilizing microbial and clinical features such as GTV, we developed a predictive model with strong performance, achieving an AUC of 88% in the training cohort and 86.9% in the test cohort.

In recurrent NPC patients undergoing re-irradiation, the incidence of necrosis affecting the nasopharyngeal mucosa, soft tissues, and bone can reach as high as 30–50% [26]. PRNN often extend into the parapharyngeal space, where the lack of a bony barrier leaves the internal carotid artery vulnerable to dissection or rupture once eroded. One study reported that among 81 patients with PRNN involving the internal carotid artery, 40 (49.4%) succumbed to sudden massive hemorrhage [27]. Therefore, the occurrence of PRNN significantly reduces the survival prognosis of patients with NPC. Clinicians urgently need to improve treatment protocols for PRNN and identify methods to identify high-risk patients to reduce the incidence of PRNN.

Previous studies have identified several clinical indicators, such as age, pathology, history of diabetes, and initial T stage, along with biochemical markers including hemoglobin, albumin, and C-reactive protein, as independent predictors of PRNN[28]. Additional research has highlighted other risk factors for PRNN following re-irradiation, including female sex, pre-existing necrosis, a cumulative prescription dose of ≥ 145.5 Gy, and a recurrent tumor volume of ≥ 25.38 cm^3^ [29]. Furthermore, recent advancements in deep learning have enabled the use of multimodal information fusion technology to integrate features from multi-sequence MRI images with patient radiotherapy dosimetry, significantly enhancing the predictive accuracy of PRNN [13]. Building on these findings, our study systematically collected all potential clinical risk factors for PRNN following re-irradiation. However, in this study, we found that only the GTV of the recurrent tumor emerged as a significant independent risk factor, as all patients received nearly identical cumulative radiation doses recommended by the clinical guidelines in recent years. This finding aligns with clinical practice, where patients with excessively large recurrent tumor volumes are often not recommended re-irradiation.

Among patients with PRNN, localized nasopharyngeal infections are commonly observed, and local irrigation with saline or antibiotics has proven effective in alleviating necrosis [30]. This suggests that the relationship between PRNN and nasopharyngeal microbiota might be significantly underestimated [31]. A growing body of evidence has elucidated the intricate relationship between tumors and intratumoral microbiota. Garrett et al. highlighted that microbiota may influence tumor development by modulating the balance between cell proliferation and apoptosis, reprogramming the immune system, and affecting host metabolism [32]. Microbial characteristics have emerged as valuable tools in tumor research. Numerous studies have demonstrated their potential to predict patient prognosis, evaluate the efficacy of immunotherapy, and identify the risks associated with adverse reactions to radiotherapy [33]. Furthermore, growing evidence highlights the critical role of gut microbiota in mitigating radiation-induced damage, particularly in protecting the hematopoietic and digestive systems. Specific microbial taxa, such as Lachnospiraceae and Enterococcaceae, along with their associated metabolic pathways, such as the propionate and tryptophan pathways, have been shown to play pivotal roles in these protective mechanisms [34]. The severity of oral mucositis in patients with NPC following radiotherapy has been closely linked to alterations in the oral microbiota, with facultative anaerobes from the phylum Proteobacteria showing a significant association [35].

Our study provides a preliminary characterization of the intratumoral microbiota in patients with recurrent NPC, revealing that the predominant phyla were Proteobacteria, Firmicutes, and Actinobacteria. Notably, Proteobacteria, a phylum consisting entirely of Gram-negative bacteria, includes species such as *Escherichia coli* which are frequently associated with inflammation and pathogenicity [36]. Firmicutes encompasses a diverse range of species, many of which are beneficial to the host, such as lactobacilli, while others, including *Staphylococcus epidermidis* and *Staphylococcus aureus*, are recognized opportunistic pathogens [37]. These findings underscore the diversity of the tumor microenvironment and its potential implications for disease progression and therapeutic strategies.

The analysis revealed significant differences in the composition, abundance, and diversity of intratumoral microbiota between the two groups. Specifically, patients with higher microbial diversity in the nasopharynx were more prone to develop PRNN after re-irradiation. Taxonomic profiling identified 17 taxa, including *Brevundimonas*, *Caulobacteraceae*, and *Oceanospirillales*, that were enriched in the necrosis group, whereas *Corynebacterium* was predominantly enriched in the non-necrosis group. Metabolic pathway analysis further implicated glycerolipid metabolism and porphyrin, and chlorophyll metabolism as potential contributors to PRNN development.

To determine whether nasopharyngeal microbiota could predict PRNN following re-irradiation, we used 5R 16S rRNA sequencing to extract microbial features from biopsy tissues of patients with recurrent NPC. These microbial features were combined with clinical parameters (GTV) to develop two predictive models: the Microbiota Model, which is based solely on microbial features, and the GTV-Microbiota Model, which integrates both microbial features and GTV. Model performance was evaluated using a test set, and the results demonstrated that the GTV-Microbiota Model significantly outperformed both the Microbiota Model and traditional GTV-based model in predicting PRNN risk.

PRNN and its associated severe epistaxis represent some of the most severe complications of recurrent NPC following re-irradiation. These adverse events not only significantly impact patients'quality of life, but also pose Life-threatening risks in severe cases. A recent multicenter, prospective, randomized phase 3 clinical trial demonstrated that hyperfractionated IMRT significantly reduces the incidence of PRNN by delivering lower doses twice daily instead of a single higher dose [7]. Despite advances in radiotherapy techniques, preventing and mitigating PRNN remains a considerable challenge in the management of recurrent NPC. Therefore, understanding the underlying mechanisms and identifying predictive markers for PRNN are critical for improving outcomes and guiding therapeutic strategies.

Our study identified distinct microbial profiles between patients who developed PRNN and those who did not, indicating a potential association between the nasopharyngeal microbiota and PRNN risk. Further research is warranted to determine whether alterations in microbial composition contribute to PRNN development and to explore the potential of microbiota-based interventions in mitigating its occurrence. Profiling the nasopharyngeal microbiota prior to re-irradiation may serve as a valuable tool for identifying high-risk patients predisposed to PRNN. For these high-risk individuals, targeted interventions, such as precision antibiotic therapy or microbiota transplantation, could be explored to restore microbial balance, potentially mitigating PRNN risk.

Future research should prioritize the development of high-throughput screening methods combined with advanced in vivo models to identify specific microbial taxa or metabolites that contribute to PRNN. Such studies could also focus on elucidating the mechanisms by which microbial dysbiosis exacerbates radiation-induced tissue damage. In parallel, randomized controlled trials assessing the efficacy of microbiota-based interventions, such as antibiotics, probiotics, or fecal microbiota transplantation, could provide robust evidence for clinical application. By leveraging these advanced methodologies, future work has the potential to significantly reduce the incidence of PRNN and improve treatment outcomes for patients with recurrent NPC.

This study has several limitations. First, its retrospective design precluded the collection of longitudinal data, limiting our ability to assess temporal changes in the microbiota and their dynamic relationship with PRNN. Second, only three patients (2.7%) in our cohort had diabetes mellitus. This condition is associated with impaired immune function and increased susceptibility to infection, which may influence local tissue status and microbial composition. Third, antibiotic usage——a factor known to influence the composition and function of the tumor microbiome, was not accounted for, potentially affecting the tumor microenvironment and immune response. Future studies should include antibiotic history to better evaluate its impact on treatment outcomes and prognosis in patients with NPC. Lastly, the relatively low incidence of recurrent NPC and the limited number of patients opting for re-irradiation as a curative treatment constrained the sample size and may have affected the representativeness of our findings, potentially limiting the identification of the microbiota characteristics associated with PRNN.

## Conclusions

This study demonstrated that nasopharyngeal microbial diversity prior to re-irradiation was significantly higher in the necrosis group than in the non-necrosis group. Building on this finding, we developed and validated a predictive model that integrates the clinical and microbial characteristics of PRNN. The model exhibited a robust predictive performance. These results underscore its potential as a valuable tool for identifying high-risk patients and guiding clinical decision making in re-irradiation strategies. Moreover, this work lays a solid scientific foundation for the early prediction and prevention of PRNN by leveraging insights into the nasopharyngeal microbiota.

## Supplementary Information

Supplementary Material 1. Fig. S1. Comparison of follow-up duration between necrosis and non-necrosis groups. Data are presented as boxplots with individual data points overlaid. The horizontal line inside each box represents the median; box limits indicate the interquartile range; whiskers indicate 1.5×IQR; and circles represent individual patients. No significant difference was found between the two groups (Mann–Whitney U test, *p* = 0.595).

## Data Availability

The datasets analyzed during the current study are not publicly available because of the protection of individual patient privacy but are available from the corresponding author upon reasonable request.

## Abbreviations

PRNN: Post-radiation nasopharyngeal necrosis
NPC: Nasopharyngeal carcinoma
GTV: Gross tumor volume
IMRT: Introduction of intensity-modulated radiotherapy
ICA: Internal carotid artery
BMI: Body Mass Index
KPS: Karnofsky Performance Status score
WBC: White blood cell
NEUT: `Neutrophil
RBC: Red blood cell
HGB: Hemoglobin
CRP: C-reactive protein
CTV: Clinical target volume
OAR: Organs at risk
GTVnd: Gross nodal tumor volume of cervical lymph node
ASVs: Amplicon sequence variants
PCA: Principal component analysis
PCoA: Principal coordinates analysis
LDA: Linear discriminant analysis
ROC: Receiver Operating Characteristic
AUC: Area Under the Curve

## References

[1] Cao S-M, Xu Y-J, Lin G-Z, et al. Estimation of cancer burden in Guangdong Province, China in 2009. Chin J Cancer. 2015;34(12):594–601. 10.1186/s40880-015-0060-4. 26573607 10.1186/s40880-015-0060-4 PMC4647496

[2] Ng W-T, Wong ECY, Cheung AKW, et al. Patterns of care and treatment outcomes for local recurrence of NPC after definite IMRT-a study by the HKNPCSG. Head Neck. 2019;41(10):3661–9. 10.1002/hed.25892. 31350940 10.1002/hed.25892

[3] Lee AWM, Ng WT, Chan JYW, et al. Management of locally recurrent nasopharyngeal carcinoma. Cancer Treat Rev. 2019;79: 101890. 10.1016/j.ctrv.2019.101890. 31470314 10.1016/j.ctrv.2019.101890

[4] Li YQ, Tian YM, Tan SH, et al. Prognostic model for stratification of radioresistant nasopharynx carcinoma to curative salvage radiotherapy. J Clin Oncol. 2018;36(9):891–9. 10.1200/JCO.2017.75.5165. 29412781 10.1200/JCO.2017.75.5165

[5] You R, Zou X, Hua Y-J, et al. Salvage endoscopic nasopharyngectomy is superior to intensity-modulated radiation therapy for local recurrence of selected T1–T3 nasopharyngeal carcinoma – a case-matched comparison. Radiother Oncol. 2015;115(3):399–406. 10.1016/j.radonc.2015.04.024. 25987536 10.1016/j.radonc.2015.04.024

[6] Liu YP, Wen YH, Tang J, et al. Endoscopic surgery compared with intensity-modulated radiotherapy in resectable locally recurrent nasopharyngeal carcinoma: a multicentre, open-label, randomised, controlled, phase 3 trial. Lancet Oncol. 2021;22(3):381–90. 10.1016/s1470-2045(20)30673-2. 33600761 10.1016/S1470-2045(20)30673-2

[7] You R, Liu YP, Xie YL, et al. Hyperfractionation compared with standard fractionation in intensity-modulated radiotherapy for patients with locally advanced recurrent nasopharyngeal carcinoma: a multicentre, randomised, open-label, phase 3 trial. Lancet. 2023;401(10380):917–27. 10.1016/s0140-6736(23)00269-6. 36842439 10.1016/S0140-6736(23)00269-6

[8] Han F, Zhao C, Huang S-M, et al. Long-term outcomes and prognostic factors of re-irradiation for locally recurrent nasopharyngeal carcinoma using intensity-modulated radiotherapy. Clin Oncol (R Coll Radiol). 2012;24(8):569–76. 10.1016/j.clon.2011.11.010. 22209574 10.1016/j.clon.2011.11.010

[9] Hua Y-J, Chen M-Y, Qian C-N, et al. Postradiation nasopharyngeal necrosis in the patients with nasopharyngeal carcinoma. Head Neck. 2009;31(6):807–12. 10.1002/hed.21036. 19340873 10.1002/hed.21036

[10] Chang KP, Tsang NM, Chen CY, et al. Endoscopic management of skull base osteoradionecrosis. Laryngoscope. 2000;110(7):1162–5. 10.1097/00005537-200007000-00018. 10892689 10.1097/00005537-200007000-00018

[11] Yang Q, Zou X, You R, et al. Proposal for a new risk classification system for nasopharyngeal carcinoma patients with post-radiation nasopharyngeal necrosis. Oral Oncol. 2017;67:83–8. 10.1016/j.oraloncology.2017.02.012. 28351585 10.1016/j.oraloncology.2017.02.012

[12] Chen M-Y, Mai H-Q, Sun R, et al. Clinical findings and imaging features of 67 nasopharyngeal carcinoma patients with postradiation nasopharyngeal necrosis. Chin J Cancer. 2013;32(10):533–8. 10.5732/cjc.012.10252. 23816556 10.5732/cjc.012.10252 PMC3845539

[13] Lu S, Xiao X, Yan Z, et al. Prognosis Forecast of Re-irradiation for Recurrent Nasopharyngeal Carcinoma based on Deep Learning Multi-modal Information Fusion. IEEE Journal of Biomedical and Health Informatics 2023:1–12. 10.1109/JBHI.2023.3286656 10.1109/JBHI.2023.3286656 37384472

[14] Sreenath SB, Grafmiller KT, Tang DM, et al. Free tissue transfer for skull base osteoradionecrosis: a novel approach in the endoscopic era. Laryngoscope. 2023;133(3):562–8. 10.1002/lary.30315. 35920134 10.1002/lary.30315

[15] Wahl MJ. Osteoradionecrosis prevention myths. Int J Radiat Oncol Biol Phys. 2006;64(3):661–9. 16458773 10.1016/j.ijrobp.2005.10.021

[16] Wang L, Yang J, Peng S-Y, et al. Microbial etiology, susceptibility profile of postradiation nasopharyngeal necrosis patients with nasopharyngeal carcinoma. Cancer Radiother. 2020;24(2):93–8. 10.1016/j.canrad.2019.09.008. 32057645 10.1016/j.canrad.2019.09.008

[17] Du Y, Feng R, Chang ET, et al. Influence of pre-treatment saliva microbial diversity and composition on nasopharyngeal carcinoma prognosis. Front Cell Infect Microbiol. 2022;12: 831409. 10.3389/fcimb.2022.831409. 35392614 10.3389/fcimb.2022.831409 PMC8981580

[18] Zheng D, Liwinski T, Elinav E. Interaction between microbiota and immunity in health and disease. Cell Res. 2020;30(6):492–506. 10.1038/s41422-020-0332-7. 32433595 10.1038/s41422-020-0332-7 PMC7264227

[19] Gerassy-Vainberg S, Blatt A, Danin-Poleg Y, et al. Radiation induces proinflammatory dysbiosis: transmission of inflammatory susceptibility by host cytokine induction. Gut. 2018. 10.1136/gutjnl-2017-313789. 28438965 10.1136/gutjnl-2017-313789

[20] Qiao H, Tan X-R, Li H, et al. Association of intratumoral microbiota with prognosis in patients with nasopharyngeal carcinoma from 2 hospitals in China. JAMA Oncol. 2022;8(9):1301–9. 10.1001/jamaoncol.2022.2810. 35834269 10.1001/jamaoncol.2022.2810 PMC9284409

[21] Tian Y-M, Zhao C, Guo Y, et al. Effect of total dose and fraction size on survival of patients with locally recurrent nasopharyngeal carcinoma treated with intensity-modulated radiotherapy: a phase 2, single-center, randomized controlled trial. Cancer. 2014;120(22):3502–9. 10.1002/cncr.28934. 25056602 10.1002/cncr.28934

[22] Hua Y-J, Han F, Lu L-X, et al. Long-term treatment outcome of recurrent nasopharyngeal carcinoma treated with salvage intensity modulated radiotherapy. Eur J Cancer. 2012;48(18):3422–8. 10.1016/j.ejca.2012.06.016. 22835782 10.1016/j.ejca.2012.06.016

[23] Logue JB, Stedmon CA, Kellerman AM, et al. Experimental insights into the importance of aquatic bacterial community composition to the degradation of dissolved organic matter. ISME J. 2016;10(3):533–45. 10.1038/ismej.2015.131. 26296065 10.1038/ismej.2015.131 PMC4817675

[24] Nejman D, Livyatan I, Fuks G, et al. The human tumor microbiome is composed of tumor type-specific intracellular bacteria. Science. 2020;368(6494):973–80. 10.1126/science.aay9189. 32467386 10.1126/science.aay9189 PMC7757858

[25] Douglas GM, Maffei VJ, Zaneveld JR, et al. PICRUSt2 for prediction of metagenome functions. Nat Biotechnol. 2020;38(6):685–8. 10.1038/s41587-020-0548-6. 32483366 10.1038/s41587-020-0548-6 PMC7365738

[26] Sun X-S, Xiao Z-W, Liu S-L, et al. Nasopharyngeal necrosis contributes to overall survival in nasopharyngeal carcinoma without distant metastasis: a comprehensive nomogram model. Eur Radiol. 2023;33(5):3682–92. 10.1007/s00330-023-09431-4. 36735041 10.1007/s00330-023-09431-4

[27] Zou X, Wang S-L, Liu Y-P, et al. A curative-intent endoscopic surgery for postradiation nasopharyngeal necrosis in patients with nasopharyngeal carcinoma. Cancer Commun (Lond). 2018;38(1):74. 10.1186/s40880-018-0338-4. 30577735 10.1186/s40880-018-0338-4 PMC6303844

[28] Li X-Y, Sun X-S, Liu S-L, et al. The development of a nomogram to predict post-radiation necrosis in nasopharyngeal carcinoma patients: a large-scale cohort study. Cancer Manag Res. 2019;11:6253–63. 10.2147/CMAR.S197841. 31372033 10.2147/CMAR.S197841 PMC6626898

[29] Yu YH, Xia WX, Shi JL, et al. A model to predict the risk of lethal nasopharyngeal necrosis after re-irradiation with intensity-modulated radiotherapy in nasopharyngeal carcinoma patients. Chin J Cancer. 2016;35(1): 59. 10.1186/s40880-016-0124-0. 27357728 10.1186/s40880-016-0124-0 PMC4928250

[30] Xiao Y, Peng S, Tang Y, et al. Retrospective analysis of a modified irrigation method for nasopharyngeal carcinoma patients with post-radiation nasopharyngeal necrosis. Front Oncol. 2021;11: 663132. 10.3389/fonc.2021.663132. 34026642 10.3389/fonc.2021.663132 PMC8139247

[31] Huang X-M, Zheng Y-Q, Zhang X-M, et al. Diagnosis and management of skull base osteoradionecrosis after radiotherapy for nasopharyngeal carcinoma. Laryngoscope. 2006;116(9):1626–31. 16954993 10.1097/01.mlg.0000230435.71328.b9

[32] Garrett WS. Cancer and the microbiota. Science. 2015;348(6230):80–6. 10.1126/science.aaa4972. 25838377 10.1126/science.aaa4972 PMC5535753

[33] Yang L, Li A, Wang Y, et al. Intratumoral microbiota: roles in cancer initiation, development and therapeutic efficacy. Signal Transduct Target Ther. 2023;8(1):35. 10.1038/s41392-022-01304-4. 36646684 10.1038/s41392-022-01304-4 PMC9842669

[34] Guo H, Chou W-C, Lai Y, et al. Multi-omics analyses of radiation survivors identify radioprotective microbes and metabolites. Science. 2020. 10.1126/science.aay9097. 33122357 10.1126/science.aay9097 PMC7898465

[35] Zhu XX, Yang XJ, Chao YL, et al. The potential effect of oral microbiota in the prediction of mucositis during radiotherapy for nasopharyngeal carcinoma. EBioMedicine. 2017;18:23–31. 10.1016/j.ebiom.2017.02.002. 28216066 10.1016/j.ebiom.2017.02.002 PMC5405060

[36] Collins JW, Keeney KM, Crepin VF, et al. *Citrobacter rodentium*: infection, inflammation and the microbiota. Nat Rev Microbiol. 2014;12(9):612–23. 10.1038/nrmicro3315. 25088150 10.1038/nrmicro3315

[37] Kost Y, Rzepecki AK, Deutsch A, et al. Association of *Staphylococcus aureus* colonization with severity of acute radiation dermatitis in patients with breast or head and neck cancer. JAMA Oncol. 2023;9(7):962–5. 10.1001/jamaoncol.2023.0454. 37140927 10.1001/jamaoncol.2023.0454 PMC10160990

