# Supplementary figures and images for: Predictive value of nasopharyngeal microbiota for necrosis after re-irradiation in recurrent nasopharyngeal carcinoma
Source: BMC Cancer. 2025 Sep 29;25:1436. doi: 10.1186/s12885-025-14842-1 (PMC12482230; doi:10.1186/s12885-025-14842-1)

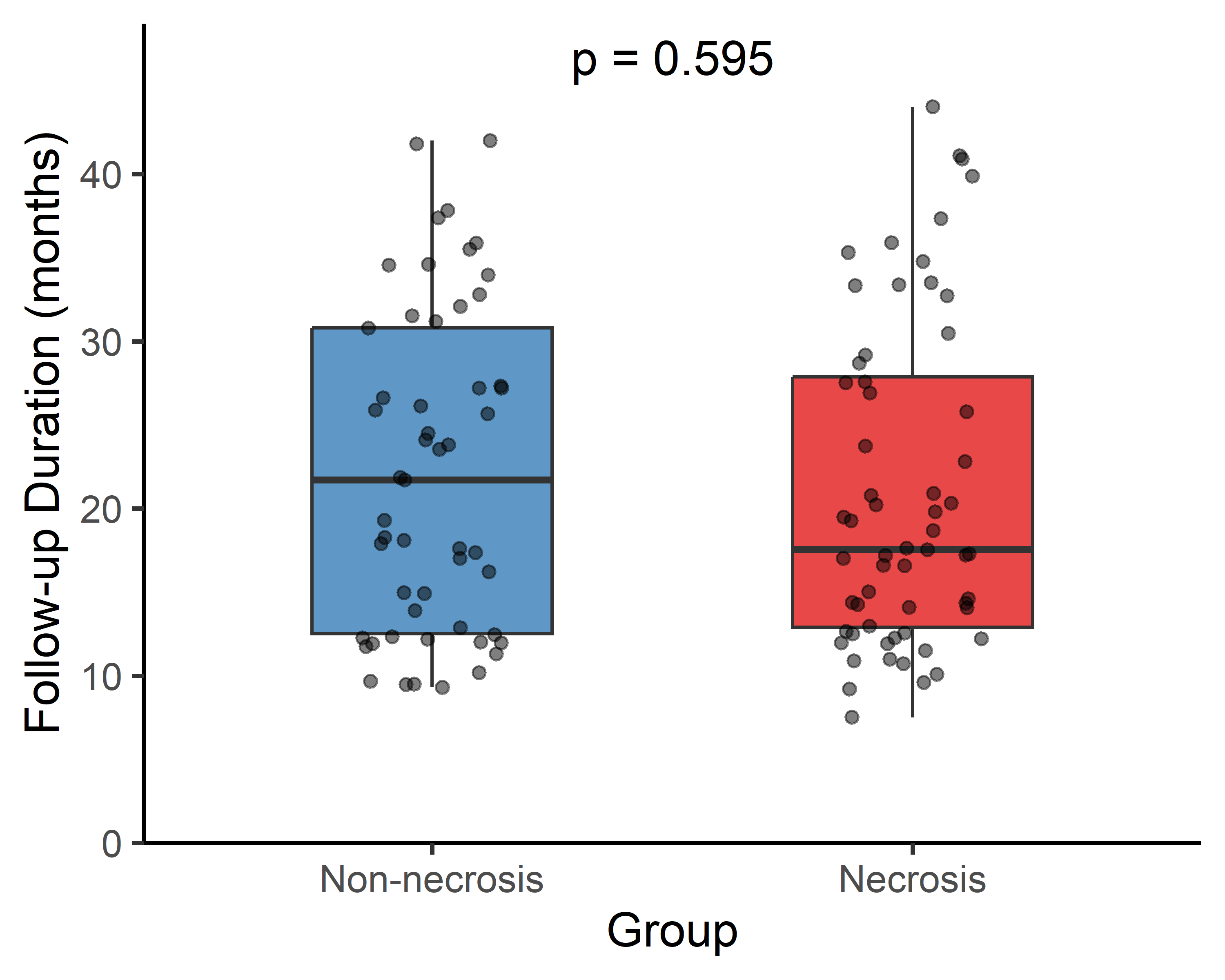

Supplement: Supplementary file 1 — Supplementary Material 1. Fig. S1. Comparison of follow-up duration between necrosis and non-necrosis groups. Data are presented as boxplots with individual data points overlaid. The horizontal line inside each box represents the median; box limits indicate the interquartile range; whiskers indicate 1.5×IQR; and circles represent individual patients. No significant difference was found between the two groups (Mann–Whitney U test, p = 0.595). [file 12885_2025_14842_MOESM1_ESM.png]
